# Clopidogrel transfer into human milk: case series – a contribution from the ConcePTION project

**DOI:** 10.3389/fphar.2025.1499243

**Published:** 2025-04-01

**Authors:** Martje Van Neste, Nina Nauwelaerts, Raf Mols, Kaytlin Krutsch, Michael Ceulemans, Anneke Passier, Anne Smits, Pieter Annaert, Karel Allegaert

**Affiliations:** ^1^ Clinical Pharmacology and Pharmacotherapy, Department of Pharmaceutical and Pharmacological Sciences, KU Leuven, Leuven, Belgium; ^2^ Child and Youth institute, KU Leuven, Leuven, Belgium; ^3^ Drug Delivery and Disposition, Department of Pharmaceutical and Pharmacological Sciences, KU Leuven, Leuven, Belgium; ^4^ Department of Obstetrics and Gynecology, InfantRisk Center, Texas Tech University Health Sciences Center, Amarillo, TX, United States; ^5^ Teratology Information Service, Netherlands Pharmacovigilance Centre Lareb’s, Hertogenbosch, Netherlands; ^6^ Research Foundation Flanders (FWO), Brussels, Belgium; ^7^ Department of Development and Regeneration, KU Leuven, Leuven, Belgium; ^8^ Neonatal Intensive Care Unit, University Hospitals Leuven, Leuven, Belgium; ^9^ BioNotus GCV, Niel, Belgium; ^10^ Department of Hospital Pharmacy, Erasmus University Medical Center, Rotterdam, Netherlands

**Keywords:** case series, breastfeeding, human milk, pharmacokinetics, cerebral vascular accident (CVA), clopidogrel, clopidogrel active metabolite (CAM), clopidogrel carboxylic acid (CCA)

## Abstract

**Introduction:**

Implementation of breastfeeding recommendations is hampered by–among others–lacking information regarding medicine safety during breastfeeding. This article describes the clinical and pharmacokinetic data of breastfeeding mothers using clopidogrel (CLP) as secondary prevention following (suspicion of) a cerebrovascular accident.

**Methods:**

A 29-year-old and 42-year-old woman were chronically treated with 75 mg CLP once daily. Human milk samples were collected at 7 and 9 months (patient 1), and at 14 months *postpartum* (patient 2). Each sampling period, two maternal blood samples as well as one infant blood sample were collected. Concentrations of CLP, clopidogrel carboxylic acid (CCA) and clopidogrel active metabolite (CAM) derivatized were analyzed using liquid chromatography with tandem mass spectrometry.

**Results:**

The average steady-state concentration in human milk was 0.96 and 7.40 ng/mL for CLP and CCA, respectively. CAM concentrations in all but two milk samples were below the limit of detection (LOD; 0.004 ng/mL). In the infant plasma sample, CCA level was 0.05 ng/mL but CLP and CAM were undetectable (CLP LOD: 0.003 ng/mL). The mean daily infant dosage (DID) was 82.3, 585.6 and 1.5 ng/kg/day for CLP, CCA and CAM, respectively, and the relative infant dose (RID) for CLP-related exposure remained well below 1%.

**Discussion:**

The estimated infant exposure to CLP and its metabolites via human milk was low in both cases. Although this low exposure was supported by the observed infant plasma concentration, additional studies should confirm CLP safety via human milk, especially considering known variable pharmacokinetics and ontogeny of metabolizing enzymes in infants.

## 1 Introduction

Exclusive breastfeeding is recommended by the World Health Organization (WHO)^1^ up to 6 months of age and partial breastfeeding with complimentary food up to 2 years of age, because of the advantages on maternal and infant health. Knowledge regarding medicine use during breastfeeding is expanding, however, insights into medicine safety and transfer to human milk is still limited. Lack of knowledge can result in uncertainty and discontinuation of breastfeeding, postponing of maternal pharmacotherapy, or off-label use of medicines.

Questions regarding the safety of clopidogrel (CLP) exposure during breastfeeding are prevalent. To put this statement in perspective, the InfantRisk Call Center^2^ in the United States explored their requests received over a 12-year period. Based on their database, 306 calls regarding CLP use and safety while breastfeeding were received during this period, of which the majority came from patients. Furthermore, they found that CLP is the fifth most prevalent reason to recommend cessation of breastfeeding from 2021–2023 (personal communication, Kaytlin Krutsch).

CLP is used as secondary prevention of atherothrombotic events, such as a myocardial infarction or a cerebrovascular accident and is considered an essential medicine by the WHO.^3^ CLP (molecular weight: 321.82 g/mol) is a prodrug that is metabolized in the liver into the inactive clopidogrel carboxylic acid (CCA) and the clopidogrel active metabolite (CAM). CAM binds irreversibly to the adenosine diphosphate (ADP) purigenic P2Y_12_ platelet receptor, which prevents platelet aggregation. CLP occurs rapidly absorbed and metabolized (oral bioavailability: 50%) as the maximum concentration (C_max_) of CAM is 30–60 min after intake. Both metabolites are highly bound to plasma proteins (98%) and the apparent volume of distribution in adults is reported to be 39.2 ± 33.5 L. CCA is formed by esterases and comprises 85% of CLP metabolism. Multiple cytochrome P450 (CYP) enzymes metabolize CLP to an intermediate metabolite 2-oxo-clopidogrel and further to CAM, mainly by CYP2C19, but also by CYP1A2, CYP2B6 and CYP3A4. CLP is excreted both in urine (50%) and in feces (46%). The half-life of CLP is circa 6 h in adults, whereas the half-life of CCA and CAM is approximately 8 h and 30 min, respectively (European Medicines Agency, 2023; Drugbank online, 2024).

The extent of metabolism from CLP to CAM is variable due to the different genotypes of the activating CYP2C19 enzyme. Four different phenotypes have been described according to genotype: ultra-rapid, extensive, intermediate and poor metabolizers. CYP2C19 has different alleles with two prominent alleles being nonfunctional (CYP2C19*2 and *3), resulting in a poor metabolizer status. The poor metabolizer phenotype is prevalent in 2% of the Caucasian population, in 4% of the Black population and in 14% of the Chinese population. Contrarily, gain-of-function alleles (CYP2C19*17) have also been reported and result in the ultra-rapid metabolizer phenotype. Pharmacogenetic tests to determine the CYP2C19 genotype are available but are not routinely recommended, even though subsequent dose-adjustment was proven as cost-effective (European Medicines Agency, 2023; Morris et al., 2022; Hertz et al., 2024).

CLP is used off-label in neonates with certain heart diseases at risk for thrombosis (e.g., after systemic-to-pulmonary artery shunt). However, dose finding has been challenging in neonates due to maturation of metabolizing enzymes, further modulated by CYP2C19 polymorphisms (Van den Anker et al., 2020). Generally, CLP intake is not recommended during pregnancy and breastfeeding (Nana et al., 2021). To our knowledge, no cases regarding CLP use during breastfeeding have been described yet. While acetylsalicylic acid is an alternative antiplatelet drug, there is no adequate information on its safety during breastfeeding either (Bell et al., 2011; Drugs and Lactation Database, 2021).

This study was conducted within the UmbrelLACT study and is a part of the Innovative Medicine Initiative (IMI) ConcePTION project^4^, intending to reduce the knowledge gap regarding medicine use and safety during pregnancy and lactation (Van Neste et al., 2024). These two case studies aimed to determine the concentration of CLP and its main metabolites in human milk and the estimated infant exposure, as well as safety data. This article was written according to the CARE guidelines and ‘Guidelines for reporting cases of medication use during lactation’ (Gagnier et al., 2013; Anderson, 2022).

## 2 Methods

This case series describes 2 European mother-infant pairs included in the UmbrelLACT study (NCT06042803), the protocol of which has been published (Van Neste et al., 2024). Shortly, both mothers provided a signed informed consent form for themselves and their infant prior to enrolment. Our study team was not involved in the two mothers’ decision to start clopidogrel treatment while breastfeeding, nor in any other aspect of their clinical care.

After inclusion, the individual patients completed a self-reporting questionnaire on maternal clinical data, such as biometry, medicine intake, and lactation-related information (Van Neste et al., 2024).

Over a period of 24 h, starting from CLP intake, human milk samples were collected at steady-state. Human milk was expressed at home using an electric pump by patient 1 and using a manual pump by patient 2 to collect the total milk volume from both breasts at each breastfeeding moment. This volume was noted and 2 samples (3 and 7 mL) were set aside in the refrigerator, in separate tubes with and without the addition of reagent 2-bromo-3′-methoxyacetophenone (MPB), respectively. This reagent was needed to stabilize the CAM by derivatization, as described for plasma (Takahashi et al., 2008). The human milk samples were stored in the patient’s refrigerator for maximum 24 h and were afterwards transferred for long-term storage.

Maternal blood samples were collected within an hour of the first and last expression session of the 24-h sampling day. After blood collection, the derivatization reagent MPB was immediately added to one of the 2 tubes. For the first blood sample of patient 2, the MPB was added too late which caused unreliable results of the derivatized CAM (CAMD) in this blood sample, so this plasma sample was excluded in further analysis. Additionally, we collected one plasma sample of the partially breastfed infant of patient 2.

On each sampling day, a standardized questionnaire on the general health outcomes of the infant was completed by both mothers. This questionnaire was repeated once again 2 months after the last sampling day (Van Neste et al., 2024).

All samples were transferred to KU Leuven, Belgium, on ice for long-term storage (−80°C). Liquid chromatography with tandem mass spectrometry (LC-MS/MS) was used for the analysis of the samples for CLP, CCA and CAM (see Supplementary Material). The lower limit of quantification (LLOQ) for CLP, CCA and CAM was 0.008, 0.38 and 0.009 ng/mL and the limit of detection (LOD) for CAM is LOD 0.005 ng/mL. Sample concentrations below the LLOQ were processed as LLOQ/2 (Beal, 2001), when calculating pharmacokinetic parameters, such as area under the curve (AUC_0–24h_) and average steady-state concentration (C_av,ss_) and infant exposure estimations, such as daily infant dosage (DID) and relative infant dose (RID). Data analysis and graphs were prepared in Excel (version 16.85).

## 3 Results

### 3.1 Case descriptions: patient information and clinical findings

#### 3.1.1 Case 1

The first mother was a 29-year-old, Caucasian woman who started CLP treatment (75 mg 1x/day) approximately 4 months *postpartum* because of an ischemic stroke, due to patent foramen ovale. Otherwise, the mother had no co-morbidities, including no liver and kidney failure. She did not use recreational drugs, nor did she smoke or drink alcohol. She followed a nut- and fish free diet. The infant was a healthy term girl and the second breastfed child of the mother. She received breastfeeding (5–7x/day) with complementary foods.

At the first sampling day at 7 months *postpartum*, the mother weighted 76.5 kg and measured 1.85 m (BMI: 22.4 kg/m^2^) and the child 8.3 kg and 69 cm, respectively. The second sampling day occurred at 9 months *postpartum*. At that time, the mother’s weight was 75 kg (BMI: 21.9 kg/m^2^) and the infant’s weight and height were 8.6 kg and 73 cm, respectively.

#### 3.1.2 Case 2

The 42-year-old, Caucasian mother started CLP intake (75 mg 1x/day) 12 months *postpartum*, due to a suspicion of a transient ischemic attack (TIA). She reported no kidney or liver problems, however, she reported to suffer from Crohn’s disease. She consumed a minimal amount of alcohol and reported no smoking or recreational drug use. No specific diet was followed. Two miscarriages were reported in her medical history. The infant was a boy, born at 41 weeks gestational age, and was the fourth child of the mother receiving breastfeeding (6–10 times daily, in combination with complementary foods).

One sampling day was scheduled for this mother-infant pair at 14 months *postpartum*. The BMI of the mother was 28.7 kg/m^2^ (83 kg, 170 cm) at that time and the biometry of the infant was ±11.2 kg and 76 cm at ±12 months *postpartum*.

### 3.2 Timeline

**FIGURE 1:**
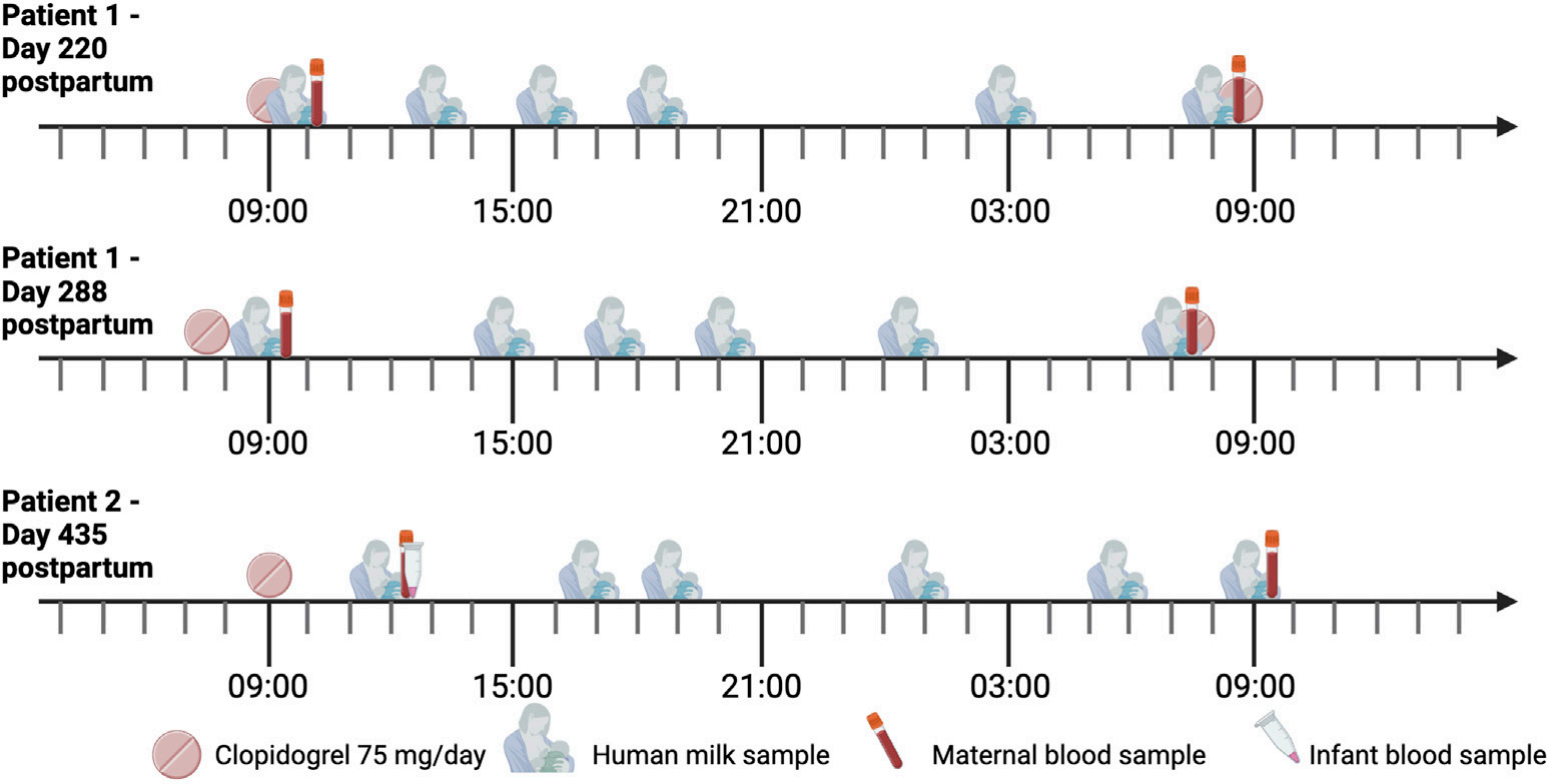
Timeline of the sampling days. Both mothers were treated with 75 mg clopidogrel once daily. Two samples were collected from each expression session. The sampling day of patient 2 was scheduled on her last day of clopidogrel intake.

The sampling moments are depicted in Figure 1.

### 3.3 Therapeutic interventions

Patient 1 used clopidogrel Mylan 75 mg film-coated tablets (Mylan, Canonsburg, Pennsylvania, United States of America) once daily after a meal on the first sampling day and before the meal on the second day. CLP may be used with or without food. However, CLP combined with a (fatty) meal had a 57% decrease in CAM Cmax, but an unchanged CAM AUC_0–24h_ (FDA, 2024). On the first sampling day, she combined this compound with ‘compleet zwanger + omega-3 visolie’ (i.e., multivitamin capsules with minerals and fish oil, 1x/day, company Davitamon), vitamin D (2800, IE, capsules, 1x/week, Davitamon) and occasionally acetaminophen (1,000 mg). The next sampling day, she did not take vitamin D anymore and had started using acetylsalicylic acid (80 mg, gastro-resistant tablets, 1x/day, Orifarm). No interactions were expected when combined with these supplements or co-medication.

Patient 2 was treated with clopidogrel oral 75 mg 1x/day which was not taken in combination with food. Co-medication was octasa (mesalazine/mesalamine, 4.8 g, 6 film-coated tablets, 1x/day, Tillotts Pharma), pentasa (mesalazine/mesalamine, 1g, suppository, 1x/day, Ferring pharmaceuticals), lansoprazole (15 mg, capsule, 1x/day, Teva), folic acid (5 mg, tablet, 1x/day, Accord), rennie (calcium carbonate, 1,000 mg, 2 tablets, 1x/day, Bayer), cod liver oil (omega-3 Fish Oil Plus, 885 mg fish oil and 308 mg omega-3,1 capsule/day, Seven Seas) and KSM-66 ashwaghanda plus (160 mg, 2 capsules, 1x/day, Wild Nutrition). Intake of these co-medications or supplements are not expected to interact with CLP or its metabolites, except for lansoprazole which decreases CLP effect by diminishing CYP2C19 activity (Collet et al., 2014).

### 3.4 Follow-up and outcomes

#### 3.4.1 Maternal health

Bruising was reported as adverse event by both patients in the self-reporting standardized questionnaire, but patient 1 reported this only when used in combination with acetylsalicylic acid. Bleeding, e.g., bruising or haematomas, is described in the Summary of Product Characteristics as a common side effect of CLP (frequency: ≥1/100 to <1/10) (European Medicines Agency, 2023).

#### 3.4.2 Infant health

Data on infant growth, hospital admissions, adverse effects and medication use were reported via a standardized questionnaire completed by both mothers. Patient 1 reported no adverse events, hospitalization or medicine use by her infant. Patient 2 reported infant allergic enterocolitis to peas and red food dye (E122), but this was already present before maternal CLP intake. Her infant received Wellbaby Multi-vitamin drops (Vitabiotics).

#### 3.4.3 Maternal samples collection

Over 3 sampling days (7–14 months *postpartum*) of 24 h each, starting from CLP intake, steady-state human milk samples and maternal blood samples were collected. The results are outlined in Table 1 and pharmacokinetic parameters or infant exposure are reported as mean (total range) throughout this paper, unless mentioned otherwise.

**TABLE 1:** Summary of the collected samples and the concentrations of clopidogrel (CLP), clopidogrel carboxylic acid (CCA) and derivatized clopidogrel active metabolite (CAMD).

Time of sampling (hh:mm)	Time between sampling and last medicine intake (hours)	Sample type^†^	Concentration (ng/mL)		
			CLP	CCA	CAM
*Patient 1 – Day 220 postpartum*					
10:10	1.17	Plasma	1.21	1,649	3.629
13:00	4	Milk	1.11	22.44	0.004*
15:50	6.83	Milk	0.36	10.10	0.004*
18:30	9.5	Milk	0.15	6.53	0.004*
03:08	18.13	Milk	0.03	2.37	0.004*
08:05	23.08	Milk	0.03	1.50	0.004*
08:32	23.5	Plasma	0.05	45.25	0.007
*Patient 1 – Day 288 postpartum*					
08:50	1.38	Milk	10.46	36.32	0.181
09:15	1.8	Plasma	1.95	1,009	3.530
14:45	7.3	Milk	2.19	9.36	0.004*
17:22	9.92	Milk	1.36	6.13	0.004*
20:09	12.7	Milk	0.96	3.88	0.004*
00:28	17.02	Milk	0.78	2.81	0.004*
07:02	23.58	Milk	0.42	1.25	0.004*
07:32	24.08	Plasma	0.13	37.86	0.057
*Patient 2 – Day 435 postpartum*					
11:50	2.78	Milk	1.69	26.96	0.004*
12:15	3.2	Plasma	0.96	385.1	NA
16:45	7.7	Milk	0.09	6.00	0.004*
19:02	9.98	Milk	0.05	4.56	0.004*
01:01	15.97	Milk	0.02	3.36	0.004*
05:40	20.62	Milk	0.02	1.79	0.004*
09:04	24	Milk	0.03	1.43	0.004*
09:28	24.4	Plasma	0.06	36.32	0.018

^†^Plasma samples refer to maternal plasma samples. *Samples under the lower limit of quantification (LLOQ) were reported as LLOQ/2. NA: this result was unreliable due to issues during sample handling. CLP: clopidogrel; CCA: clopidogrel carboxylic acid; CAM: clopidogrel active metabolite.

#### 3.4.4 Human milk and plasma pharmacokinetics

**FIGURE 2:**
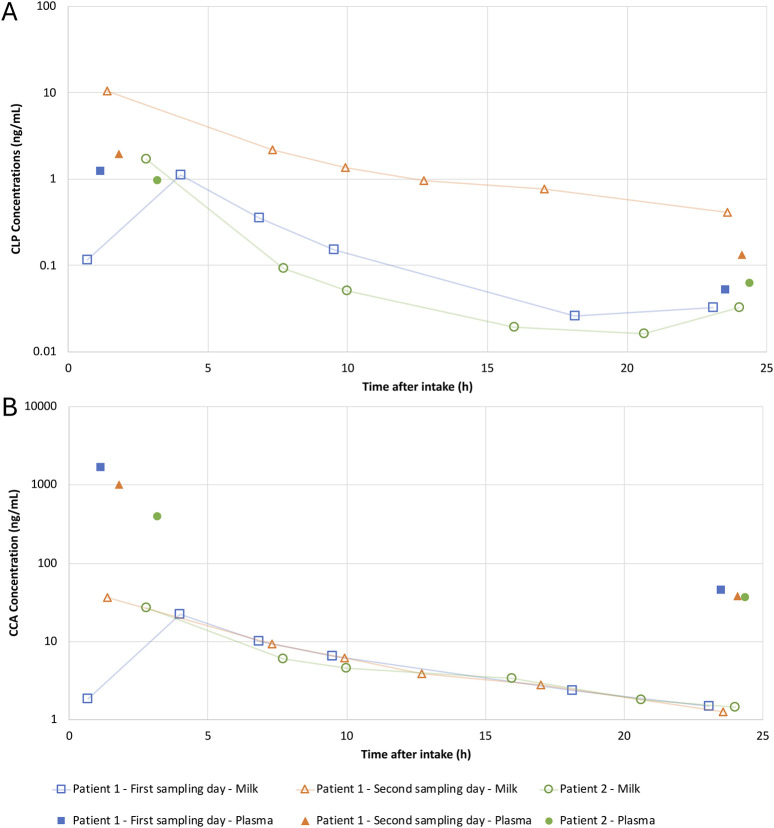
Steady-state human milk and plasma concentration-time profiles of clopidogrel (CLP ) **(A)** and the inactive metabolite carboxylic acid (CCA) **(B)** during intake of 75 mg once daily. Levels from patient 1 at day 220 (Δ) and from patient 2 at day 435 postpartum (•) in human milk and plasma.

The concentrations of CLP and CCA are provided in Figure 2. All CAM concentrations in the human milk samples were below the LOD (0.005 ng/mL), except for the first milk samples of the sampling days of patient 1 (0.028 and 0.181 ng/mL). The mean AUC_24h_ in human milk were 21.20, 162.7 and 0.28 ng*h/mL, and the mean C_av,ss_ were 0.96, 7.40 and 0.01 ng/mL for CLP, CCA and CAM, respectively.

As only two plasma concentrations were determined per sampling day, we were unable to calculate a plasma AUC_24h_. Therefore, the mean plasma AUC_24h_ in non-postpartum patients using clopidogrel 75 mg once daily (CLP 5.07 ng*h/mL, CCA 11077 ng*h/mL and CAM 11.3 ng*h/mL), without specifics on metabolizer phenotype, was extracted from Karazniewicz-Łada et al. (2014) (Karaźniewicz-ŁAda et al., 2014).

#### 3.4.5 Milk-to-Plasma (M/P) ratio

The mean M/P ratio calculated with milk AUC_24h_ from these patients and plasma AUC_24h_ from literature (as referenced in the previous paragraph) was 4.2, 0.01 and 0.03 for CLP, CCA and CAM, respectively (Equation 1) (Karaźniewicz-ŁAda et al., 2014).

$$M/P\text{ratio}=\frac{{\text{AUC}}_{\text{human}\;\text{milk}}}{{\text{AUC}}_{\text{plasma}}}$$ (1)

The M/P ratio for single time points can be calculated using the concentration of the paired plasma-milk samples (first and last expression sessions) from Table 1. However, this method is less accurate than the M/P ratio based on AUC.

#### 3.4.6 Impact of CYP2C19 phenotypes

To improve the estimated AUC-based M/P ratio for different CYP2C19 phenotypes, the data set of Danielak (2014) with individual data on different CYP2C19 phenotypes of 45 patients (male and female) using 75 mg CLP once daily was consulted (Dorota, 2014). The comparative data set consisted of concentration-time profiles of CLP, CCA and CAM reported over 24 h at steady state.

**FIGURE 3:**
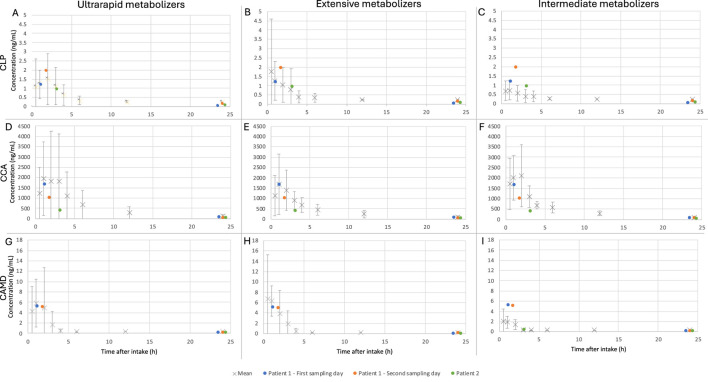
Comparison of different CYP2C19 phenotypes to this case series for clopidogrel (CLP) **(A–C)**, clopidogrel carboxylic acid (CCA) **(D–F)** and derivatized clopidogrel active metabolite (CAMD) **(G–I)** concentrations. The mean (X) steady-state plasma concentrations were generated for the different types of metabolizers after intake of 75 mg CLP once daily, based on individual data from Danielak (2014). The lower limit of quantification for their data set was 0.25, 50 and 0.25 ng/mL for CLP, CCA and CAMD, respectively. The concentrations of this case series (•) are visually compared to the concentrations of the different phenotypes: ultrarapid metabolizers **(A, D, G)**, extensive metabolizers **(B, E, H)** and intermediate metabolizers **(C, F, I)**.

The mean and standard deviation for each phenotype was visually compared to the measured concentrations of CLP, CCA and CAM of the patients included in our study. As the phenotype was not determined for these patients, both patients were considered to be extensive metabolizers based only on visual evaluation (Figure 3). When calculating the M/P ratio with the plasma AUC of extensive metabolizers (7.06, 7,640 and 20.3 ng*h/mL for CLP, CCA and CAM respectively), the means of the three sampling days were 3.0, 0.02 and 0.01 for CLP, CCA and CAM, respectively.

#### 3.4.7 Infant plasma sample

One plasma sample of the breastfed infant of patient 2 was collected and analyzed. The levels of CLP and CAM were under the LOD. Therefore, the LLOQ was used to calculate the exposure as a ‘worst case’ scenario. The infant plasma concentrations of CLP, CCA and CAM were ≤0.008, 0.049 and ≤0.009 ng/mL, respectively. Compared to the maternal plasma concentration, the relative infant exposure using a single time point (Equation 2) via human milk was ≤0.8% and 0.01% for CLP and CCA, respectively.

$$Relative\;infant\;exposure\;\left(\%\right)=\frac{Infant\;plasma\;concentration\;\left(\frac{ng}{mL}\right)}{Maternal\;plasma\;concentration\;\left(\frac{ng}{mL}\right)}*100$$ (2)

The relative infant exposure for CAM was not available, due to the unreliable maternal plasma sample. Similarly to the maternal CYP2C19 genotype, the genotype of the infants was also unknown.

#### 3.4.8 Infant exposure

The exposure of a breastfed infant over a 24-h period was estimated using the DID (Equation 3), where n is the number of milk expression sessions during the study period and milk volume the noted volume of each expression session.

$$DID\;\left(\frac{\frac{ng}{kg}}{day}\right)=\sum_{i=1}^{n}\left(\frac{{Milk\;Concentration}_{\;i}\left(\frac{ng}{L}\right)*{Milk\;Volume}_{i}\;\left(L\right)}{Infant\;weight\;\left(kg\right)}\right)$$ (3)

The mean DID of CLP, CCA and CAM were 82.9 (9.4–211.7), 585.6 (280.1–765.1) and 1.5 (0.2–3.0) ng/kg/day, respectively. The DID is highly dependent on human milk intake of the infant. As both infants in this case series received complementary foods as well, the milk intake was low in both cases.

Additionally, the RID compares the daily dosage of the infant via human milk to the weight corrected daily maternal dose (Equation 4).

$$\text{RID }\left(\%\right)=\frac{DID\;\left(\frac{\frac{ng}{kg}}{day}\right)}{Daily\;Maternal\;Dose\;\left(\frac{ng}{day}\right)/Maternal\;Weight\;\left(kg\right)}*100$$ (4)

The mean RID for CLP was 0.008% and ranged from 0.001% to 0.021%. To take the overall CLP exposure into account, we calculated the RID based on the molar amounts of CLP and both metabolites, resulting in a RID of 0.071% (0.033%–0.101%).

The DID via breastfeeding was compared to a daily therapeutic infant dosage of 1 mg/kg/day^5^ and 0.2 mg/kg/day (Li et al., 2008) clopidogrel to determine the Relative Infant Therapeutic Dose (RID_therapeutic_) (Equation 5).

$${\text{RID}}_{therapeutic}\;\left(\%\right)=\frac{DID\;\left(\frac{\frac{ng}{kg}}{day}\right)}{Daily\;Therapeutic\;Infant\;Dosage\;\left(\frac{\frac{ng}{kg}}{day}\right)}*100$$ (5)

The mean RID_therapeutic_ for CLP was 0.008% (0.001%–0.021%) for a dosage of 1 mg/kg/day and 0.041% (0.005%–0.106%) for 0.2 mg/kg/day. When considering all CLP-related exposure, mean RID_therapeutic_ is 0.070% (0.030%–0.101%) and 0.348% (0.151%–0.506%) for 1 mg/kg/day and 0.2 mg/kg/day, respectively. No standard dosing is recommended in infants and children by regulatory agencies, and its use is not favored due to efficacy concerns (European Medicines Agency, 2023; FDA, 2024).

Lastly, the DIDs for CLP and its metabolites were re-calculated for an exclusively breastfed child based on the C_av,ss_ and a human milk intake of 150 mL/kg/day, and 200 mL/kg/day, to estimate the potential risk of clopidogrel exposure via breast milk in early infancy (Equation 6).

$$DID\left(\frac{\frac{ng}{kg}}{day}\right)=Average\;Steady‐State\;Milk\;Concentration\left(\frac{ng}{L}\right)*Infant\;Milk\;Intake\left(\frac{\frac{L}{kg}}{day}\right)$$ (6)

This resulted in mean DIDs of 143.6, 1,109.7 and 1.9 ng/kg/day for CLP, CCA and CAM, respectively, for exclusively breastfed infants (150 mL/kg/day). Considering 200 mL/kg/day as daily milk intake, the mean DIDs for CLP, CCA and CAM were 191.5, 1,479.6 and 2.6 ng/kg/day, respectively. When calculating the exposure to CLP and its metabolites combined, the mean DID (RID) was 1,305 (0.13%) and 1740 (0.18%) ng/kg/day for an intake of 150 mL/kg/day and 200 mL/kg/day, respectively.

## 4 Discussion

This case series is to our knowledge the first to provide observations on the human milk concentration of clopidogrel (CLP) and its main metabolites, the estimated infant exposure, and the reported health outcomes of the breastfed infants.

The infant exposure to maternal CLP intake was determined in the plasma of 1 breastfed infant and was low. Similarly, no infant adverse events were reported as of 2 months after the last sampling day. Furthermore, the estimated RID was lower than 0.2%, even when pooling CLP and its metabolites and using a higher daily milk intake volume of 200 mL/kg/day to mimic early infancy intake. These results are far below the arbitrary RID cut-off value of 10% (Anderson, 2018). Even though this cut-off was defined without empiric evidence, the observed RIDs are highly unlikely to cause any pharmacological effect in the breastfed infant, as supported by the absence of short-term adverse outcomes of the infants (Anderson, 2018). Furthermore, comparing the DID to the therapeutic doses administered to infants, the amount of infant exposure to CLP through human milk was confirmed to be low (0.008%–0.041%)^6^ (Li et al., 2008).

The levels of CAM are below the LOD in human milk, except for two human milk samples. Clopidogrel is a P-glycoprotein (P-gp/*ABCB1*) substrate, but this efflux pump transporter of the blood-milk barrier is described as downregulated during lactation (Sangkuhl et al., 2010; Ahmadzai, 2014). Additionally, the transporter multi-drug resistance-associated protein 3 (MRP3/*ABCC3*) might have an influence on clopidogrel exposure during breastfeeding (Luchessi et al., 2017). To the best of our knowledge, no other transporter mechanisms were found for clopidogrel in literature. Furthermore, the low half-life contributes to the low CAM level. While CLP and CCA have a half-life of 6 and 8 h, the half-life of CAM is only 30 min (European Medicines Agency, 2023; Drugbank online, 2024).

The concentrations of CLP in plasma and human milk samples were higher during the second sampling day of patient 1 compared to the first sampling day (Figure 2). This increase was not observed in the measured metabolites, neither in plasma nor human milk. This difference might be explained by interindividual variability or the concomitant intake of acetylsalicylic acid during the second sampling day. Interestingly, drug-drug interactions between proton pump inhibitors and CLP have been described (as in the case for patient 2), for which combination with lansoprazole is favorable but still results in a reduction in CYP2C19 activity and anti-thrombotic effect (European Medicines Agency, 2023; Collet et al., 2014). However, no interactions between CLP and acetylsalicylic acid have been identified yet (Serbin et al., 2016).

The enzyme activity in infants is not yet mature (ontogeny), which means that conversion of CLP into CAM in the infant is expected to be minimal. More specifically, there is only 20%–25% of CYP2C19 expression levels of adults at birth and 40%–50% of adult levels at 1 year of age (Van den Anker et al., 2020). In case of CYP3A4, a high variability in activity is reported during the first 12 months of life. Its activity increases from 8% of adult activity at birth to 43% from 3 months to 1 year of life and to 108% from 1 year to 15 years of age (Caruthers and Dorsch, 2008). Furthermore, the role of CYP3A5 and CYP3A7 in the metabolism of CLP in infants has not yet been explored. As the concentration of CLP is so low, we do not expect any adverse health effects, even in case of the higher CLP level when combined with acetylsalicylic acid.

The role of CYP2C19 is crucial in the formation of CAM, which means that the genotype of this enzyme plays an essential role in the activation of CLP as well. More specifically, poor metabolizers have a reduced exposure to CAM, which might cause a reduction in platelet responsiveness to CLP. On the other hand, ultrarapid metabolizers activate CLP more rapidly. This CYP2C19 polymorphism is subsequently an explanation for the variability seen in the effect of CLP (Van den Anker et al., 2020; Karaźniewicz-ŁAda et al., 2014; Karaźniewicz-Łada et al., 2012). However, in this case series the 2 patients were assumed to be extensive metabolizers based on their plasma levels corresponding to this phenotype, whereas the levels visually deviated from the intermediate or poor metabolizer type (Dorota, 2014). The maternal poor metabolizer phenotype should result in higher concentrations of CLP in maternal plasma and milk. This maternal phenotype would theoretically result in the highest infant systemic exposure after breastfeeding and poses the highest risks for the infant. However, since the dose ingested by the infant is estimated to be very low irrespective of the maternal polymorphism and infant conversion of CLP to CAM is expected to be minimal because of ontogeny, we do not expect a clinically significant effect of either the maternal or infant CYP2C19 metabolizer phenotype.

Some limitations were identified while conducting this study. First, since human milk sampling was performed by the patients at home, it is impossible to be certain whether the patients followed the sample instructions perfectly. Second, this specific study was complicated by the extra sample handling needed to stabilize CAM. Furthermore, our UmbrelLACT protocol describes human milk expression by an electric pump, according to the FDA guidance (FDAb). An electric pump was not available during the sampling day of patient 2, so human milk was expressed by a manual breast pump. Nevertheless, an electric pump can overestimate the human milk volume and fraction of lipid content due to complete emptying of the breasts (Anderson and Sauberan, 2016). As this study does not allow to determine the AUC in maternal plasma to calculate the M/P ratio, the M/P ratio was calculated using plasma AUC from non-breastfeeding patients from literature. As relevant characteristics of their physiology, such as body composition and enzyme activity, changes during pregnancy and lactation, the M/P ratio based on literature might differ from the real value (Van Neste et al., 2023). Moreover, CAM levels in human milk were almost all under the LOD. This implies that the AUC for CAM is very low and could not be accurately determined. Even though this is a limitation of this study, it indicates that the active component of CLP is only minimally present in human milk, emphasizing the estimated low risks of CLP intake during breastfeeding. Furthermore, the toxicity of CCA in infants has not yet been explored. Except for a slight increase in neurologic adverse events (i.e., seizure and stroke) in infants using clopidogrel (0.2 mg/kg/day) compared to placebo (7.8% vs. 2.3%, p < 0.001), clopidogrel and its metabolites are well-tolerated in infants (Wessel et al., 2013). Due to the low CCA concentrations in human milk, no toxic effects are expected from CCA. Last, the infants in this case series were over 6 months old which implies already more mature enzyme activity and were both already receiving complementary foods. Therefore, these results are less representative for medicines exposure during the first weeks or months of life.

This is the first paper that quantified CLP exposure of breastfeeding infants and provides relevant data to support shared-decision making in clinical practice. As highlighted by the numbers from the InfantRisk Call Center (unpublished data), the quantification of CLP and its metabolites in human milk is highly relevant as many clinical requests have been received regarding this compound. Discontinuation of breastfeeding had been advised in case of CLP use (European Medicines Agency, 2023; FDA, 2024), however, infant exposure seems to be low according to this first study. As breastfeeding itself has many benefits for mother and child, the low exposure is an argument pointing at compatibility of maternal CLP use and breastfeeding for these cases.

More research is required to assess the impact of genetic polymorphisms on the exposure through human milk and to confirm our results. Additionally, PBPK models of CLP and its metabolites are recommended to predict the exposure in breastfeeding infants by simulations, as only few cases or milk samples are needed for this approach (Nauwelaerts et al., 2023). These PBPK models could be extrapolated to explore challenging situations, such as different maturation of enzymes in infants or the changing physiology in lactating women during *postpartum*. Furthermore, this approach can investigate multiple scenarios related to a broader postnatal age range and all different CYP2C19 genotypes, similarly to the CYP2D6 genotypes study (Willmann et al., 2009).

In summary, an estimated low infant exposure to CLP and its metabolites in human milk was reflected by a low infant plasma concentration. This case series found no short-term adverse events from CLP use during breastfeeding. However, these two cases likely do not fully capture the exposure during early infancy and its pharmacokinetic variability, warranting additional data collection, as well as PBPK simulations on infant exposure and safety of maternal CLP during breastfeeding.

## Data Availability

The datasets presented in this article are not readily available because of concerns regarding participant/patient anonymity. Requests to access the datasets should be directed to the corresponding author, and will be considered pending a reasonable request. Requests to access the datasets should be directed to martje.vanneste@kuleuven.be.

## Footnotes

1
https://www.who.int/health-topics/breastfeeding#tab=tab_1

2
https://infantrisk.com/

3
https://www.who.int/publications/i/item/WHO-MHP-HPS-EML-2023.02

4
https://www.imi-conception.eu/

5
https://www.kinderformularium.nl/geneesmiddel/29061/clopidogrel

6
https://www.kinderformularium.nl/geneesmiddel/29061/clopidogrel
