# Supplementary material for: Clopidogrel transfer into human milk: case series – a contribution from the ConcePTION project
Source: Front Pharmacol. 2025 Apr 1;16:1499243. doi: 10.3389/fphar.2025.1499243 (PMC11996768; doi:10.3389/fphar.2025.1499243)
Supplement: Supplementary file 1 [file DataSheet1.docx]

Supplementary Material

# Bio-analysis method

Plasma and breast milk samples were analyzed after protein precipitation by reversed-phase high performance liquid chromatography (RP-HPLC) with tandem mass spectrometry (MS-MS) detection (Acquity H-class UPLC and Xevo TQ-S micro, Waters, Milford, MA, USA).

The protein precipitation was performed as follows: 100 µL of sample was added to 900 µL acetonitrile containing 5nM internal standard (13C6-clopidogrel, Alsachim, Illkirch Graffenstaden, France). Samples were then centrifuged for 5 minutes at 21000g at 4°C. Supernatant was transferred to a clean test tube and evaporated to dryness under a gentle stream of air. After drying, samples were redissolved in 200 µL mobile phase from which 150 µL was transferred into a micro HPLC vial. Lastly, 5 µL was injected in the liquid chromatograpy tandem mass spectrometry (LCMS-MS) system.

In brief, separation was performed using a Kinetex XB-C18 column (2.6 µm, 2.1 x 50 mm; Phenomenex, Utrecht, The Netherlands) held at 35°C. Methanol (solvent A) and 0.1% formic acid in water (solvent B) were used as eluents at a flow rate of 0.5 mL/min. Gradient elution was performed as follows: 45% of solvent A during 0.5 min, increase of solvent A to 70% in 0.5 min, 70% of solvent A during 1.5 min, immediate increase of solvent A to 95%, 95% of solvent A during 1 min, immediate decrease to 45% A and finally 1 min 45% solvent A to re-equilibrate the column to the starting conditions, resulting in a total runtime of 4.5 minutes. Clopidogrel carboxylic acid (CCA) eluted at 0.48 min, clopidogrel (CLP) and the internal standard 13C6-clopidogrel eluted at 1.88 min and clopidogrel active metabolite derivative (CAMD) eluted at 2.40 min.

MS-MS was carried out with a heated electrospray ionization (HESI) source in positive ionization mode on a Xevo TQ-S micro mass detector (Waters, Milford, MA, USA) with following parameters: capillary voltage 1000 V, cone voltage 30 V, source temperature 150°C, desolvation temperature 600°C, desolvation gas flow 800 L/Hr, cone gas flow 50 L/Hr. The following mass transitions for the detection of the different compounds were used: CCA m/z 308.4 🡪 198.3 (collision energy 14V), CLP m/z 322.3 🡪 212.3 (collision energy 15V), 13C6-clopidogrel m/z 328.3 🡪 218.3 (collision energy 15V) and CAMD m/z 504.4 🡪 155.3 (collision energy 40V).

Calibration curves were made on the day of the analysis by serial dilution in plasma or breast milk. Data were analyzed using the TargetLynx software.

For CCA the method was proven to be linear, accurate and precise over the range of 10000 to 1.22nM. For CLP and CAMD the range was 50 to 0.02nM. This converted to a lower limit of quantification (LLOQ) of 0.008, 0.376 and 0.010 ng/mL for CLP, CCA and CAMD, respectively. The limit of detection (LOD) for CLP and CAMD were 0.003 and 0.005 ng/mL, respectively.
